# Supplementary material for: DNA damage sensitivity of SWI/SNF-deficient cells depends on TFIIH subunit p62/GTF2H1
Source: Nat Commun. 2018 Oct 4;9:4067. doi: 10.1038/s41467-018-06402-y (PMC6172278; doi:10.1038/s41467-018-06402-y)
Supplement: Supplementary file 1 — Supplementary Information [file 41467_2018_6402_MOESM1_ESM.pdf]

Supplementary Information

DNA damage sensitivity of SWI/SNF-deficient cells depends on TFIIH subunit p62/GTF2H1

Cristina Ribeiro-Silva[1], Özge Z. Aydin[1,2], Raquel Mesquita-Ribeiro[3], Jana Slyskova[1], Angela Helfricht[1], Jurgen A. Marteijn[1], Jan H.J. Hoeijmakers[1], Hannes Lans[1,*], Wim Vermeulen[1,*]

1. Department of Molecular Genetics, Oncode Institute, Cancer Genomics Center Netherlands, Erasmus MC, 3015 GE, Rotterdam, The Netherlands

2. Current address: Molecular Biology and Genetics Department, Koç University, Istanbul, Turkey

3. School of Life Sciences, University of Nottingham, NG7 2UH, Nottingham, United Kingdom

* Correspondence and requests for materials should be addressed to:

HL (w.lans@erasmusmc.nl) or WV (w.vermeulen@erasmusmc.nl)

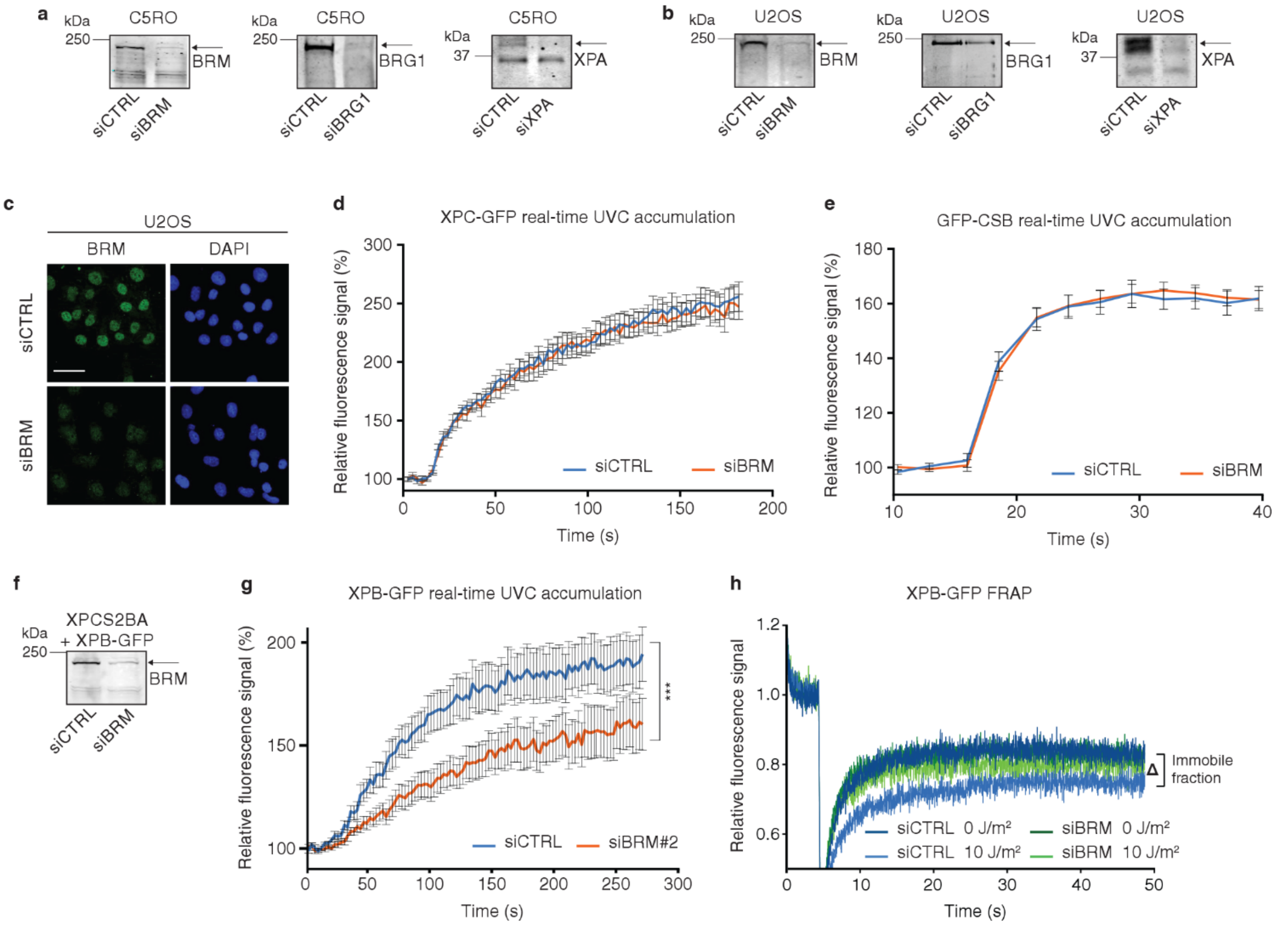

Supplementary Figure 1. BRM is required for recruitment of XPB but not of GG- and TC-NER damage sensor proteins XPC and CSB. (**a**,**b**) Immunoblot analysis of total cell lysates demonstrating siRNA-mediated downregulation of BRM, BRG1 and XPA in (**a**) C5RO (related to Fig. 1a,b) and (**b**) U2OS cells (related to Fig. 1c,d). The same total amount of protein from each cell lysate was loaded in each lane. (**c**) Immunofluorescence images demonstrating BRM siRNA-mediated depletion in U2OS cells (related to Fig. 1e,f). Scale bar: 50 µm. (**d**) XPC-GFP and (**e**) GFP-CSB accumulation at LUD, induced with a 266 nm UV-C laser, measured in real time by confocal imaging. Pre-damage relative fluorescence intensity was set to 100% (t=0). (**d**) XP4PA with stable XPC-GFP expression were treated with control (CTRL) or BRM siRNAs. Representative results of three independent experiments with similar results (mean & S.E.M., at least 10 cells per condition in each experiment). (**e**) CS1AN cells with stable GFP-CSB expression were treated with control (CTRL) or BRM siRNAs (mean & S.E.M. of at least 10 cells per condition). (**f**) Immunoblot analysis of total cell lysates demonstrating siRNA-mediated downregulation of BRM in XPCS2BA patient cell lines complemented with XPB-GFP, referring to Fig. 2a,b. The same total amount of protein from each cell lysate was loaded in each lane. (**g**) XPB-GFP accumulation at LUD, induced with a 266 nm UV-C laser, measured in real time by confocal imaging. Pre-damage relative fluorescence intensity was set to 100% (t=0). XPCS2BA cells with stable expression of XPB-GFP were treated with control (CTRL) and BRM siRNAs. Mean & S.E.M. of at least 10 cells per condition, P< 0.0001, relative to siCTRL. (**h**) FRAP analysis of XPB-GFP in mock treated or UV-irradiated (10 J/m^2) XPCS2BA cells transfected with control

(CTRL) or BRM siRNAs. XPB-GFP was bleached in a strip across the nucleus and fluorescence recovery was measured over 50 s and normalized to pre-bleach. The immobile XPB-GFP fraction depicted in Fig. 2c was calculated after subtracting the bleach-depth intensities, by dividing the average recovered fluorescence intensity of UV-irradiated cells by the average recovered fluorescence intensity of mock treated cells, over the last 10 s of the measurements as explained in the methods. FRAP graph is the average of 3 independent experiments, with at least 10 cells measured per condition each time.

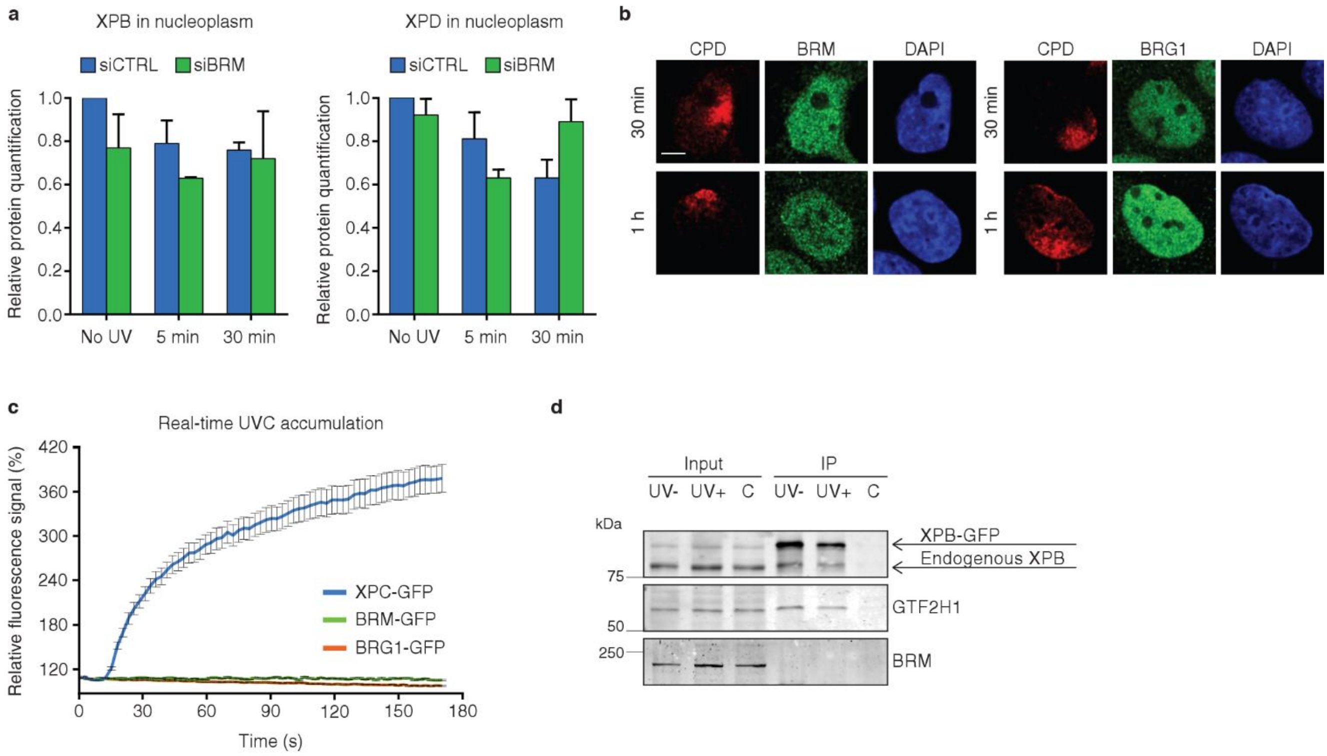

Supplementary Figure 2. BRM and BRG1 are not localized to sites of UV damage. (**a**) Relative quantification of the immunostaining of soluble (nucleoplasm) XPB and XPD (shown in Fig. 2d) from U2OS cells treated with control (CTRL) or BRM siRNAs before and after (5 and 30 min) UV-C irradiation (20 J/m^2). Mean & S.E.M. of two independent experiments. (**b**) Immunofluorescence showing absence of recruitment of BRM and BRG1 to LUD in U2OS cells. Cells were fixed 30 min and 1 h after inducing LUD with UV-C irradiation (60 J/m^2) through a microporous membrane (8 µm). UV lesions were marked with staining against CPD (red channel). Scale bar: 5 µm. (**c**) XPC-GFP (in XP4PA), BRM-GFP and BRG1-GFP (in U2OS) accumulation at LUD, induced with a 266 nm UV-C laser, measured in real time by confocal imaging. Pre-damage relative fluorescence intensity was set to 100% (t=0). Mean & S.E.M. of at least 15 cells per condition. (**d**) Immunoblot analysis of input and GFP immunoprecipitation (IP) from XPB-GFP expressing XPCS2BA cells, 30 min after mock (UV-) or global UV-C (20 J/m^2; UV+) treatment and from non-treated XPCS2BA control cells, without XPB-GFP (C). Samples were analyzed with antibodies against XPB, GTF2H1 and BRM.

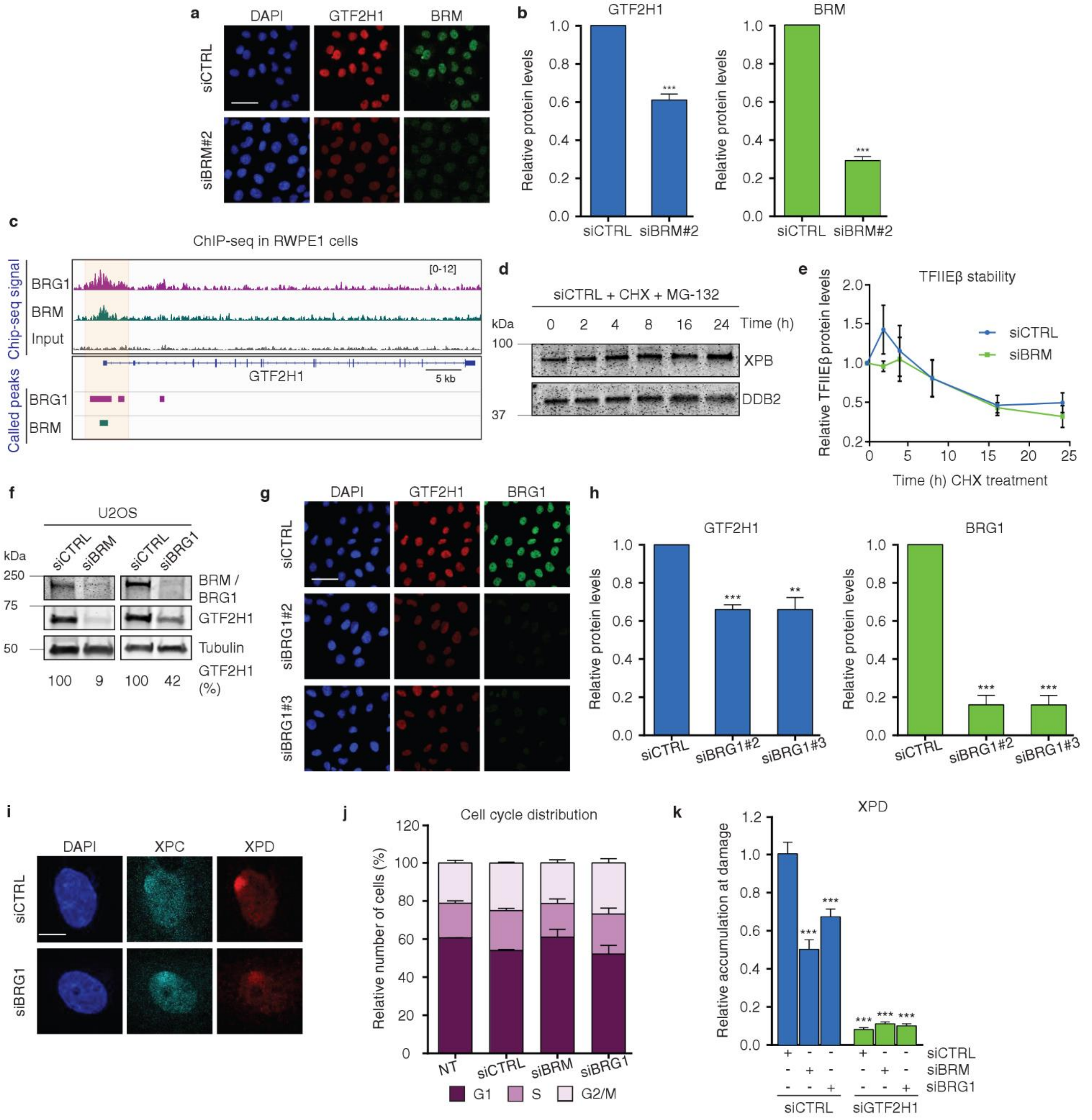

Supplementary Figure 3. Lower GTF2H1 expression after BRM or BRG1 knockdown affects TFIIH stability and function. (**a**) Immunofluorescence of GTF2H1 and BRM protein levels in U2OS cells treated with control (CTRL) or BRM siRNA RNA. For BRM, an independent siRNA (BRM#2) was used to exclude off-target effects. Scale bar: 50 µm. (**b**) Quantification of GTF2H1 and BRM fluorescence intensity, shown in (a). Total levels of GTF2H1 and BRM were normalized to control (CTRL) siRNA, set to 1.0 (mean & S.E.M. of three independent experiments, with > 110 cells per sample per experiment). ***P < 0.001, relative to siCTRL. (**c**) BRG1 and BRM co-occupancy of GTF2H1 promotor in RWPE1 cells. Re-analysis of published BRG1 and BRM ChIP-seq data from RWPE1 cells (overexpressing lncRNA SCHLAP1) in which ChIP-seq signal density (top) and respective

peaks (bottom) illustrate BRG1 (purple) and BRM (green) enrichment at the promoter of GTF2H1 (GEO accession GSE114392). Promoter region is highlighted in light orange, signal density in reads per million. (**d**) XPB stability was analyzed in U2OS cells treated with control (CTRL) siRNA and with 100 µM cycloheximide (CHX) to inhibit protein synthesis. Cells were pretreated with 50 µM of proteasome inhibitor MG-132, 45 min prior to CHX addition. DDB2 immunostaining was used as loading control. Representative immunoblots of two independent experiments with similar results. (**e**) Quantification of TFIIEβ protein levels normalized to DDB2 in time after addition of cyclohexamide (CHX) in U2OS cells treated with control (CTRL) or BRM siRNAs (as shown in Fig. 3d). The total amount of TFIIEβ in whole cell lysates was set to 1.0 at t=0 in each condition. Mean & S.E.M. of two independent experiments. (**f**) Immunoblots of BRM, BRG1 and GTF2H1 protein levels from whole cell lysates of U2OS cells treated with non-targeting (CTRL), BRM or BRG1 siRNAs. Tubulin was used as a loading control to normalize quantified GTF2H1 protein levels, indicated below the blot (normalized to siCTRL, set to 100%). (**g**) Immunofluorescence of GTF2H1 and BRG1 protein levels after BRG1 siRNA-mediated depletion with two additional independent siRNAs (BRG1#2, BRG1#3) in U2OS cells, to exclude off-target effects. Scale bar: 50 µm. (**h**) Quantification of GTF2H1 and BRG1 fluorescence intensity, shown in (g). Total levels of GTF2H1 and BRG1 were normalized to control (CTRL). Mean & S.E.M. of three independent experiments with > 110 cells per sample per experiment. **P< 0.01, ***P< 0.001, relative to siCTRL. (**i**) Immunofluorescence of XPD recruitment (red channel) to LUD marked by XPC (cyan channel) in U2OS cells treated with control (CTRL) or BRG1 siRNAs. U2OS cells were fixed 30 min after local UV-C irradiation (60 J/m^2) through a microporous membrane (8 µm) Scale bar: 5 µm. (**j**) Knockdown of BRM or BRG1 does not affect cell cycle distribution. U2OS cells were transfected with the indicated siRNAs or mock treated (NT). The average percentage of cells in G1 (dark purple), S (lighter purple) and G2/M (light pink) phase is presented. Mean & S.E.M. of at least two independent experiments. (**k**) Quantification of XPD recruitment to UV-lesions within 30 mins after UV-C irradiation (60 J/m^2) in U2OS cells treated with control (CTRL), BRM, BRG1 and GTF2H1 siRNAs. Relative accumulation of XPD at LUD (over nuclear background) in each condition was normalized to cells treated only with siCTRL, in which nuclear background was set as 0 and maximal signal at LUD set to 1.0 (>47 cells per sample, mean & S.E.M.). ***P< 0.001, relative to cells treated only with siCTRL.

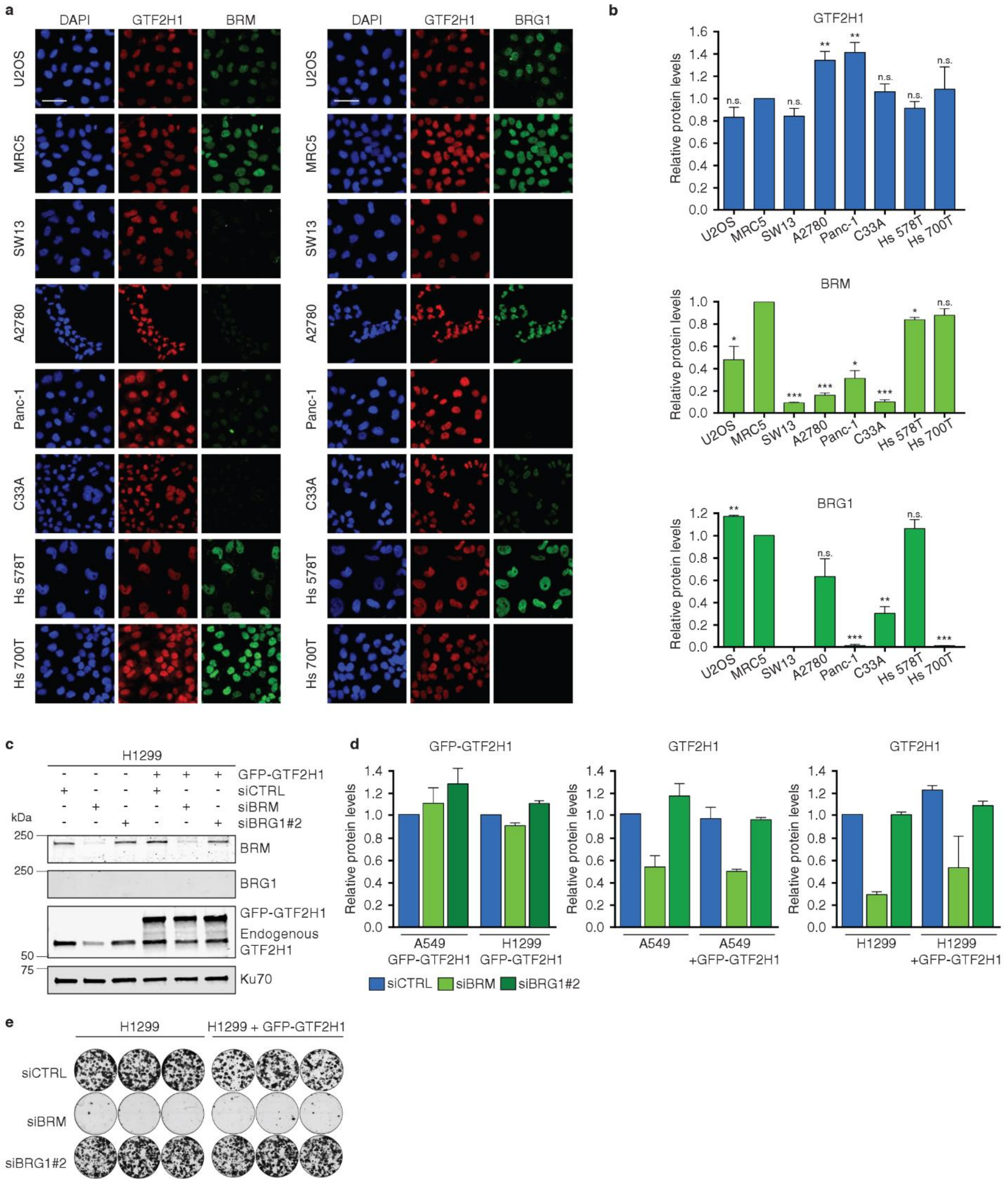

Supplementary Figure 4. GTF2H1 expression in SWI/SNF-deficient cancer cell lines. (**a**) Immunofluorescence analysis of GTF2H1, BRM and BRG1 levels in SWI/SNF-deficient cancer cell lines SW13 (adrenal cortex), A2780 (ovarian), Panc-1 (pancreatic), C33A (cervix), Hs 597T (breast) and Hs 700T (pancreatic). As reference, U2OS (osteosarcoma) and MRC5 (human fibroblasts) were used. Scale bar: 50 µm. (**b**) Quantification of GTF2H1, BRM and BRG1 fluorescence intensity, shown in (a). Total protein levels were

normalized to MRC5, set to 1.0. Mean & S.E.M. of four (GTF2H1) and two (BRM, BRG1) independent experiments with > 100 cells per sample per experiment. *P< 0.05, **P< 0.01, ***P< 0.001, relative to MRC5. n.s., non-significant. (**c**) Whole cell lysate of H1299 cells with and without stable expression of GFP-GTF2H1, treated with control (CTRL), BRM or BRG1 (BRG1#2) siRNAs, were analyzed by immunoblotting against BRG1, BRM and GTF2H1. Ku70 was used as loading control. (**d**) Relative quantification of ectopic GFP-GTF2H1 and endogenous GTF2H1 levels in H1299 (depicted in c) and A549 (depicted in Fig. 5c) cells, with and without stable expression of GFP-GTF2H1, treated with control (CTRL), BRM or BRG1 (BRG1#2) siRNAs. GFP-GTF2H1 and GTF2H1 levels were normalized to Ku70 and to siCTRL, set to 1.0 (mean & S.E.M. from two independent experiments). **(e)** H1299 cells, with or without stable expression of GFP-GTF2H1 were seeded 48 h after transfection with control (CTRL), BRM or BRG1 (BRG1#2) siRNAs, in triplicate, at a density of 750 cells per well and grown for 12 before fixation and staining.

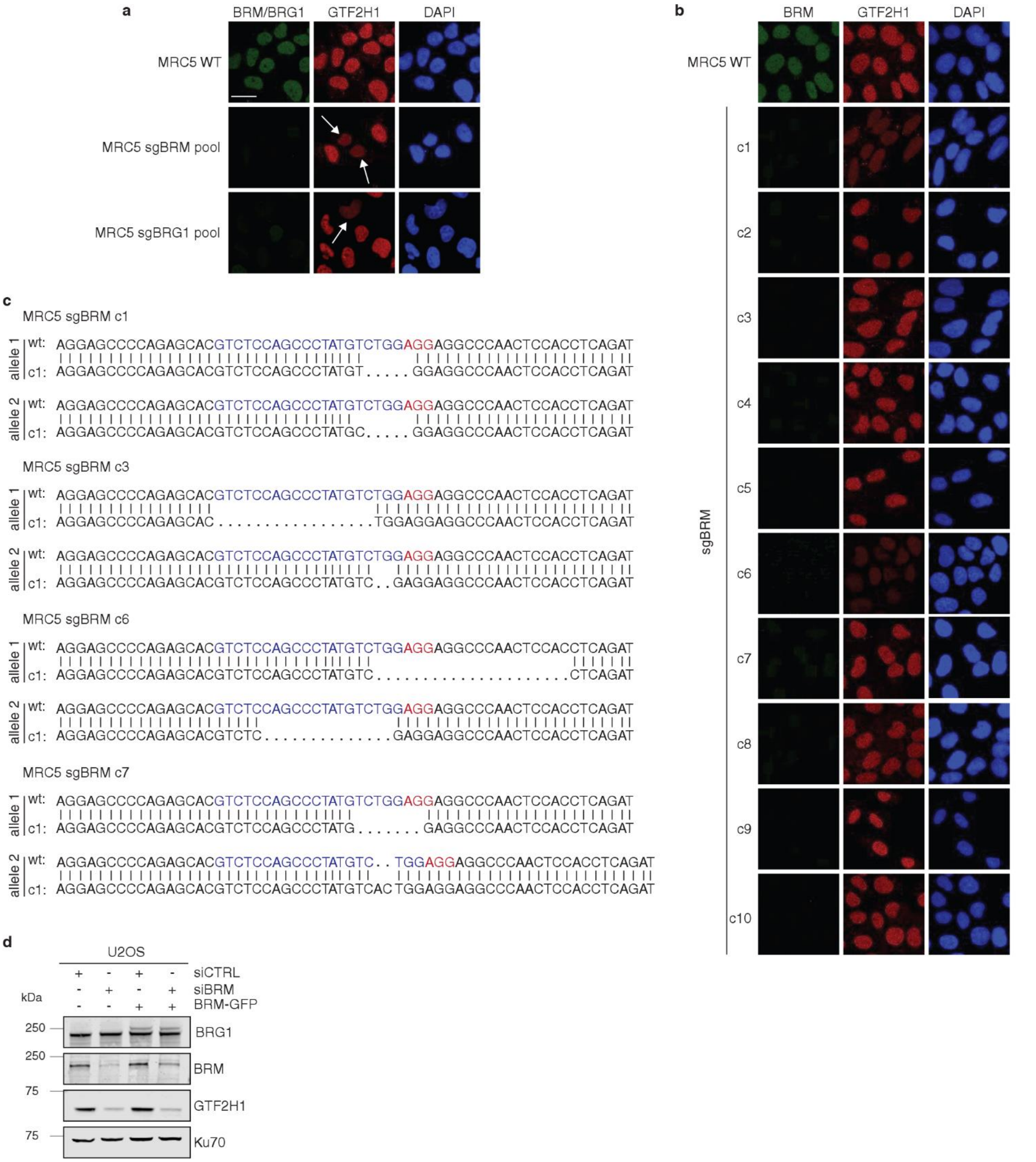

Supplementary Figure 5. Cells with permanent loss of BRM or BRG1 can restore GTF2H1 expression levels. (**a**) Immunofluorescence analysis of GTF2H1 levels in MRC5 WT cells and heterogeneous pool of sgBRM or sgBRG1 transfected cells. Arrows depict cells that have retained low GTF2H1 levels. To verify the knockouts, specific antibodies against BRM or BRG1 were used (green channel). Scale bar: 25 µm. (**b**) Immunofluorescence analysis of BRM and GTF2H1 levels in MRC5 WT and single BRM knockout (sgBRM) clones. (**c**) Sequence of the sgBRM target region in MRC5 BRM knockout clones c1, c3, c6 and c7. Blue indicates the sgBRM target

sequence. Red indicates the PAM sequence. All deletions (in alleles of c1, c3, c6 and c7) and the insertion (in one of the alleles of c7) are predicted to severely truncate BRM. (**d**) Immunoblot analysis of GTF2H1 levels in U2OS cells without and with stable ectopic expression of BRG1-GFP, treated with control (CTRL) or BRM siRNAs. Immunoblots were stained against GTF2H1, BRM, BRG1 and Ku70 as loading control.

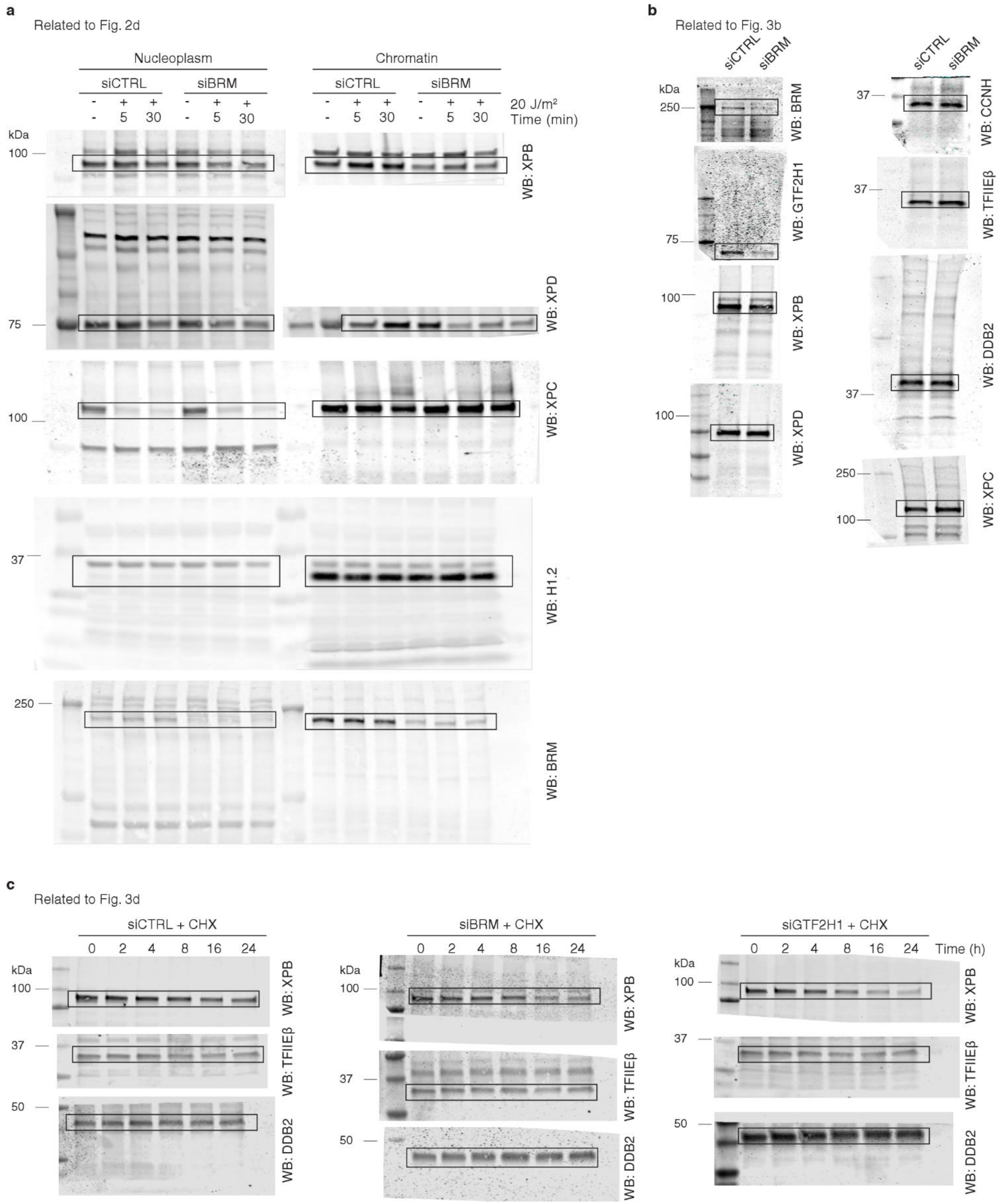

Supplementary Figure 6. Full-size immunoblot scans shown in Fig. 2d (**a**), Fig. 3d (**b**) and Fig. 3d (**c**).

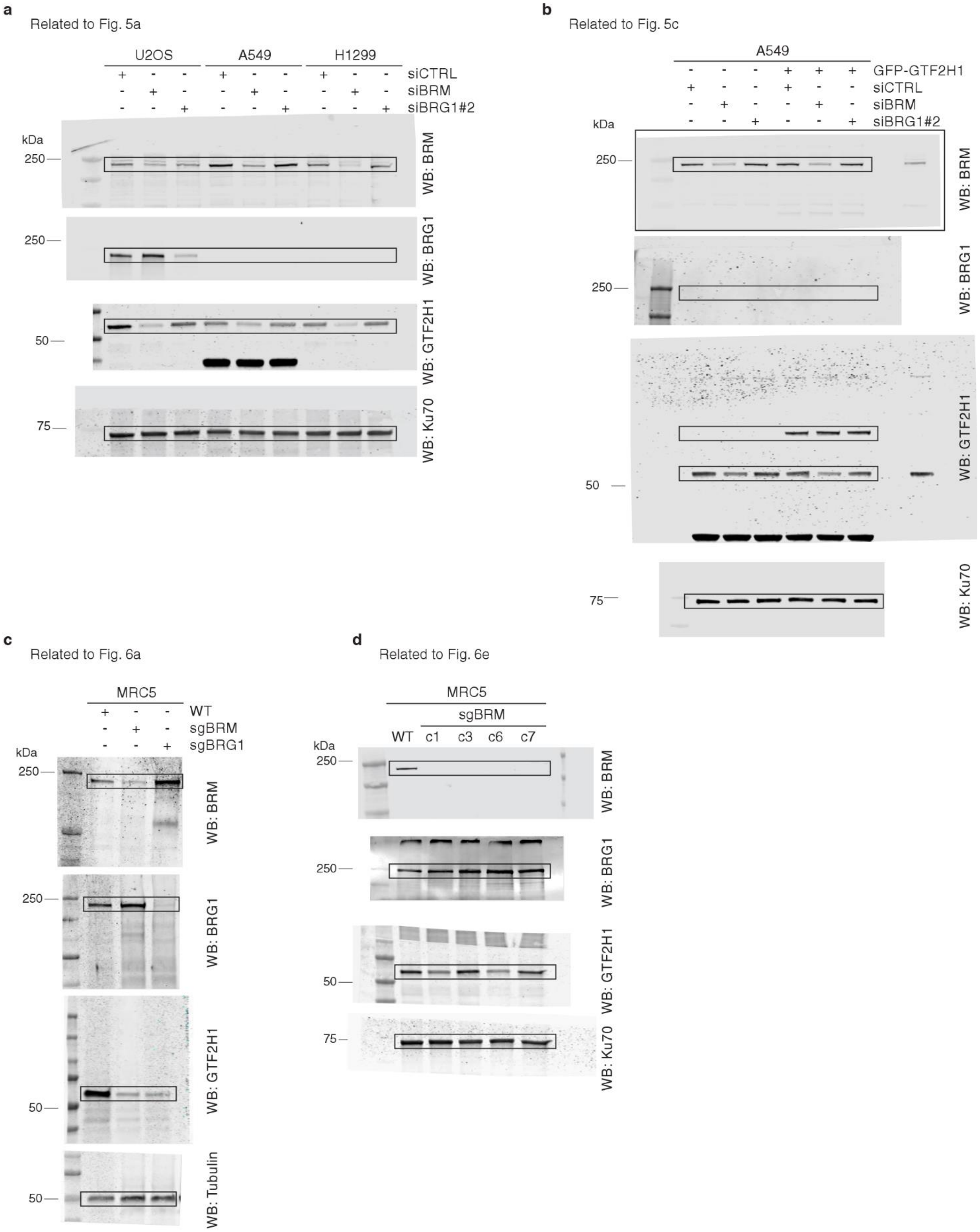

Supplementary Figure 7. Full-size immunoblot scans shown in Fig. 5a (**a**), Fig. 5c (**b**), Fig. 6a (**c**) and Fig. 6e (**d**).

Supplementary Table 1. Primary Antibody list and working dilutions.

Source, Reference	Antibody	Dilutions	
		**Immunoblotting**	**Immunofluorescence**
Abcam, ab181136	DDB2	1/1000	1/1000
Bethyl, A301-121A	XPC	1/2000	1/2000
Santa Cruz, sc-293	XPB	1/1000	1/1000
Abcam, ab54676	XPD	1/1000	1/150
Abcam, ab55199	GTF2H1	1/500	1/500
Sigma, WH0002965M1	GTF2H1	1/500	1/500
Novus Biologicals, NBP2-38556	GTF2H1	1/500	1/200
Santa Cruz, sc-853	XPA	1/250	1/50
Santa Cruz, sc-136153	XPF	N.A.	1/100
MBL international, TDM-2	CPD	N.A.	1/1000
Abcam, ab15597	BRM	1/800	1/250
Santa Cruz, sc-6450	BRM	N.A.	1/200
Sigma Aldrich, B8184	BRG1	1/3000	1/1000
Abcam, ab110641	BRG1	1/10000	1/500
2D4 clone	CCNH	1/1000	N.A.
Abcam, ab17677	Histone H1.2	1/1000	N.A.
Abcam, ab187143	TFIIEβ	1/1000	N.A.
Santa Cruz, sc-1487	Ku70	1/1000	N.A.
Santa Cruz, sc-17789	Ku70	1/1000	N.A.
Sigma Aldrich, B512	Tubulin	1/10000	N.A.

Supplementary Table 2. Secondary Antibody list and working dilutions.

Source, Reference	Antibody	Dilutions	
		**Immunoblotting**	**Immunofluorescence**
Sigma, sab4600215	Anti-rabbit, CF IRDye 770	1/10000	N.A.
Sigma, sab4600200	Anti-rabbit, CF IRDye 680	1/10000	N.A.
Sigma, sab4600214	Anti-mouse, CF IRDye 770	1/10000	N.A.
Sigma, sab4600199	Anti-mouse, CF IRDye 680	1/10000	N.A.
Sigma, sab4600375	Anti-goat, CF IRDye 770	1/10000	N.A.
Invitrogen, A11034	Anti-rabbit, Alexa Fluor 488	N.A.	1/1000
Invitrogen, A21429	Anti-rabbit, Alexa Fluor 555	N.A.	1/1000
Invitrogen, A21207	Anti-rabbit, Alexa Fluor 594	N.A.	1/1000
Invitrogen, A21072	Anti-rabbit, Alexa Fluor 633	N.A.	1/1000
Invitrogen, A11001	Anti-mouse, Alexa Fluor 488	N.A.	1/1000
Invitrogen, A21424	Anti-mouse, Alexa Fluor 555	N.A.	1/1000
Invitrogen, A21467	Anti-goat, Alexa Fluor 488	N.A.	1/1000

Supplementary Table 3. List of primers used for RT-qPCR.

Gene	Sense primer	Antisense Primer	Amplicon size (bp)
CCNH	5’-TCACCCCAGGATAATAATGCTCA-3’	5’-CAGTATCTGTTCAAGTGCCTTCT-3’	136
CDK7	5’-GGAGCCCCAATAGAGCTTATACA-3’	5’-TCCACACCTACACCATACATCC-3’	97
GTF2H1	5’-GACCTTGTTGTGAGTCAAGTGA-3’	5’-CCTGCTTATGATTGGATGTGGAA-3’	100
GTF2H2	5’-CGTATGGGATTTCCTCAGCAC-3’	5’-AGCCTCCTAATGTAAGCCCTG-3’	115
GTF2H3	5’-GAATGGCAGACTTGGAGACTTC-3’	5’-GCAAAGTTTCTGTATGTTGACCC-3’	176
GTF2H4	5’-ACCCCATTTTCCGCCAGAAC-3’	5’-CGGCGTACTTGTCAAGGGAG-3’	126
GTF2H5	5’-AAGACATTGATGACACTCACGTC-3’	5’-GGGAAAAAGCATTTTGGTCCATT-3’	96
MNAT1	5’-GGTTGCCCTCGGTGTAAGAC-3’	5’-AGTTGCTCTTTCTGAGTGGAGT-3’	160
ERCC3	5’-CTAACTGCCTACTCCTTGTATGC-3’	5’-TCCATAGCTGACAGTACACAACT-3’	141
ERCC2	5’-AGAAGGTGATTGAAGAGCTTCG-3’	5’-ACCTCAGGGTGAATACACAAGT-3’	121
GAPDH	5’-AAGGTGAAGGTCGGAGTCAA-3’	5’-ACCATGTAGTTGAGGTCAATG-3’	125
