# Supplementary material for: DNA damage sensitivity of SWI/SNF-deficient cells depends on TFIIH subunit p62/GTF2H1
Source: Nat Commun. 2018 Oct 4;9:4067. doi: 10.1038/s41467-018-06402-y (PMC6172278; doi:10.1038/s41467-018-06402-y)
Supplement: Supplementary file 3 — Description of Additional Supplementary Files [file 41467_2018_6402_MOESM3_ESM.pdf]

Description of Additional Supplementary Files

File Name: Supplementary Data 1

Description: Individual data points of immunofluorescence (from Fig. 1a,c,f, 3f, 4b, 6d,i and Supplementary Fig. 3b,h,k, 4b) and FRAP (from Fig. 2c) experiments for presentation of data distribution
